# Comparison of Prophylactic Versus Reactive Tube Feeding Approaches on Weight Loss and Unplanned Hospital Admissions in Patients with Head and Neck Cancer Receiving Chemoradiotherapy

**DOI:** 10.3390/curroncol33010005

**Published:** 2025-12-21

**Authors:** Teresa Brown, Louise Cooney, David Smith, Louise Elvin-Walsh, Eliza Kern, Suzanne Ahern, Bena Brown, Ingrid Hickman, Sandro Porceddu, Lizbeth Kenny, Brett Hughes

**Affiliations:** 1 Dietetics & Food Services, Royal Brisbane and Women’s Hospital, Brisbane, QLD 4029, Australiasuzanne.ahern@health.qld.gov.au (S.A.); 2 School of Human Movement & Nutrition Science, University of Queensland, Brisbane, QLD 4072, Australia; 3 Dietitian Senior Research Assistant and Equity in Cancer Care Coordinator, Division of Cancer Services, Princess Alexandra Hospital, Brisbane, QLD 4102, Australia; louise.cooney@health.qld.gov.au; 4 Mercy Lab, Pasadena, CA 91109, USA; 5 Nutrition and Dietetics, Queen Elizabeth II Jubilee Hospital, Brisbane, QLD 4108, Australia; louise.elvin-walsh@health.qld.gov.au; 6 Southern Queensland Centre of Excellence in Aboriginal and Torres Strait Islander Primary Health Care, Metro South Health, Brisbane, QLD 4077, Australia; bena.brown@health.qld.gov.au; 7 School of Public Health, Faculty of Health, Medicine and Behavioural Sciences, University of Queensland, Brisbane, QLD 4006, Australia; 8 ULTRA Team, Clinical Trial Capability, Faculty of Health, Medicine and Behavioural Sciences, The University of Queensland, Brisbane, QLD 4072, Australia; i.hickman@uq.edu.au; 9 Department of Radiation Oncology, Peter MacCallum Cancer Centre, Melbourne, VIC 8006, Australia; sandro.porceddu@petermac.org; 10 School of Medicine, University of Queensland, Brisbane, QLD 4072, Australiabrett.hughes@health.qld.gov.au (B.H.); 11 Cancer Care Services, Royal Brisbane and Women’s Hospital, Brisbane, QLD 4029, Australia

**Keywords:** head and neck cancer, chemoradiotherapy, tube feeding, enteral nutrition, gastrostomy, nasogastric tube

## Abstract

The study’s aim was to compare the unplanned admission rates and nutrition outcomes in patients with head and neck squamous cell cancer (HNSCC) receiving chemoradiotherapy at two different hospitals with different nutrition support approaches. Hospital Site A used prophylactic tube feeding and Site B used reactive tube feeding. Consecutive HNSCC patients receiving chemoradiotherapy with curative intent over six months in 2015 were eligible for this prospective comparative cohort study. Only patients who were classified as at high nutrition risk using validated guidelines were included. Patients’ weight was recorded at the start, end, and 4–6 weeks post treatment to determine percentage weight loss outcomes. Unplanned hospital admissions (for medical or nutrition related reasons) and associated length of stay (LOS) were collected throughout and up to 1-month post treatment. In total, 88 patients were included in the study (site A *n* = 58; site B *n* = 30). The mean age was 60 years, 86–90% were male, and predominantly had oropharyngeal cancer. There was no statistical difference between the groups for percentage weight loss at any timepoint, rates of unplanned nutrition related admissions, or LOS. Stepwise logistic analysis showed that being of an older age was predictive of having an unplanned nutrition-related admission. In summary, there was no difference in the rate of unplanned admissions or percentage weight loss for patients with HNSCC managed under the prophylactic versus reactive tube feeding approach. Decision making regarding the choice of feeding tube should be made in consultation with the patient and the multidisciplinary team.

## Simple Summary

Patients with head and neck cancer undergoing chemoradiotherapy can have significant difficulties with eating and drinking, and often require tube feeding. There is controversy in the medical literature regarding the best of form of tube feeding—either a prophylactically placed feeding tube prior to treatment (usually a gastrostomy) or a feeding tube placed during treatment when necessary (usually a nasogastric tube). This study compares these two approaches to nutrition support at two different tertiary hospitals in Brisbane, Australia. The outcomes compared include weight loss during and post treatment, and the frequency of unplanned hospital admissions. There were no statistical differences seen between hospitals; however, clinically important differences in weight loss outcomes were seen, with the prophylactic approach having less critical weight loss during and post treatment. This can improve nutritional status and has been associated with other benefits such as improved quality of life, less treatment complications, and improved treatment tolerance and survival.

## 1. Introduction

Patients with head and neck squamous cell cancer (HNSCC) are at high risk of malnutrition and swallowing dysfunction due to obstruction or the metabolic effects of the tumour itself and/or the side effects of treatment [1,2]. Malnutrition is associated with detrimental outcomes such as a reduced quality of life, poor treatment tolerance, exacerbated acute toxicities, increased treatment interruptions, increased unplanned admissions, increased risk of locoregional recurrence and cancer-related mortality, and reduced overall survival [2,3,4]. The importance of nutritional care and the optimisation of nutritional status in order to improve clinical outcomes is considered a key component of multidisciplinary care in several international evidence-based clinical practice guidelines [5,6,7]. Intensive nutrition support and counselling has also recently demonstrated associations with improved 10-year survival outcomes [8].

However, many patients with HNSCC will require enteral (tube) feeding for nutritional support when dysphagia, odynophagia, or other side effects of treatment lead to dehydration and/or malnutrition during and/or after cancer treatment. Current practice involves the options of either prophylactic gastrostomy tube placement, placed prior to treatment in anticipation of need, or reactive feeding tube (nasogastric or gastrostomy) placement when oral intake becomes unviable. Both forms of nutrition support have demonstrated superiority to oral intake alone, with improved protein and energy intakes and minimisation of weight loss; however, it is important to consider the advantages and disadvantages of each approach [9]. The aim of this multicentre study was to compare prophylactic versus reactive tube feeding approaches using prospectively collected nutrition outcome and unplanned admission data in a homogenous cohort of patients identified at diagnosis as being at a high risk of requiring a feeding tube using validated guidelines [10].

## 2. Materials and Methods

**Figure 1:**
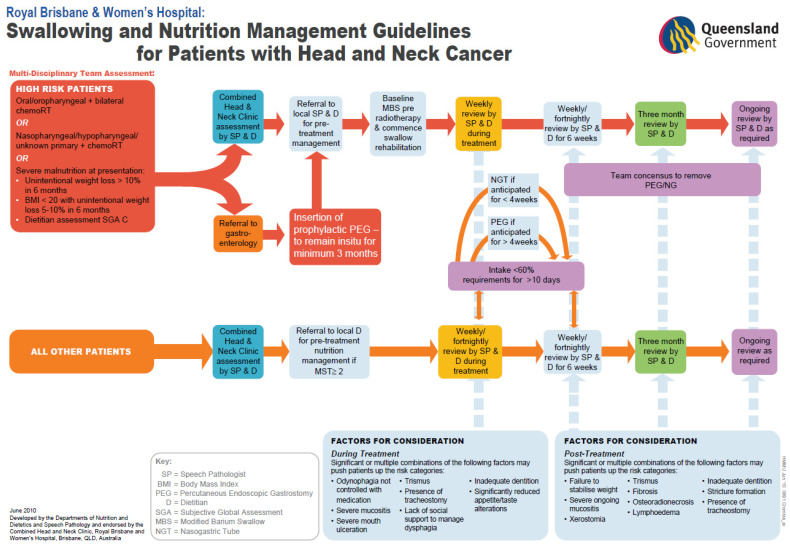
Swallowing and Nutrition Management Guidelines for Patients with Head and Neck Cancer used at Site A (reprinted/adapted with permission from Brown et al. [10]; copyright © 2013 Wiley Periodicals, Inc.). The criteria for the commencement of reactive feeding tube placement occurred when oral intake was <60% of energy requirements, with inadequate intake and no expected improvement for a total of 10 days or more.

This prospective cohort study was completed at the two largest radiation therapy sites in Queensland, Australia. The two hospitals have adopted different approaches to enteral nutrition management during HNSCC treatment, with Site A employing a prophylactic approach and Site B a reactive approach. A summary and comparison of the nutrition management strategies at each site is shown in Table 1. Patients at Site A were screened at the multidisciplinary team meeting using a validated guideline [10] (Figure 1). Patients assessed as high risk were advised to have a proactive gastrostomy feeding tube inserted prior to treatment and this was usually placed via endoscopy. If a gastrostomy was contra-indicated or declined by the patient of the treating team, then reactive tube placement (i.e., a feeding tube placed during and/or immediately after completion of treatment) was used as necessary, following the criteria in the guidelines. In contrast, Site B primarily used a reactive tube feeding approach, most commonly in the form of a nasogastric tube placement. The commencement of reactive tube feeding at Site B was a clinical decision made in conjunction with the patient, radiation oncologist, speech pathologist and dietitian, and could occur at any time during and post treatment. Although this reactive approach was the primary method of nutrition management, individual cases for prophylactic tube placement were still discussed at the multidisciplinary team (MDT) meeting, and if agreed would primarily proceed with radiological placement prior to commencement of chemoradiotherapy (CRT).

All consecutive patients who received curative intent definitive or adjuvant CRT for HNSCC at both hospitals (Site A and Site B) over a 6-month period in 2015 were screened for inclusion in this study. Only patients classified as being at a high risk of requiring a feeding tube during treatment as per the criteria in the Swallowing and Nutrition Management Guidelines (Figure 1) were included in this study, with patients who already had a feeding tube in situ at diagnosis being excluded. High-risk patients were those with oral cavity or oropharyngeal cancer plus bilateral CRT or nasopharyngeal/hypopharyngeal/unknown primary plus CRT, or those who were severely malnourished at presentation. Patients < 18 yrs of age, those treated for lymphoma or with palliative intent, or those who had not seen a dietitian during treatment were excluded. At Site A, all patients received either definitive or post-operative CRT to the head or neck area using helical intensity-modulated radiotherapy (H-IMRT). At Site B, all patients received either definitive or post-operative CRT to the head or neck area using intensity-modulated radiotherapy (IMRT). Radiotherapy to the primary tumour at both sites consisted of 5–6 fractions of 2–2.5 Gy/fraction a week, to a total dose of 56–70 Gy, and at-risk regions were treated with 50 to 50.4 Gy. Four types of systemic therapies were used across both sites. The first was cisplatin at 100 mg/m^2^ given in weeks one, four, and seven of treatment. The second was weekly cisplatin given as 40 mg/m^2^ in a weekly dose. The third therapy was cetuximab, with a loading dose of 400 mg/m^2^ followed by weekly 60 min infusions of 250 mg/m^2^. Lastly, carboplatin and 5-fluorouracil were other alternatives used. Any surgical procedures that were completed were traditional open surgical resections, as Trans Oral Robotic Surgery was not available at the time of this study.

Patients at both sites received regular dietary and swallow assessments, interventions, and education from combined dietetic and speech pathology clinics during and post treatment, as recommended in nationally endorsed evidence-based guidelines [5]. Counselling was aimed at meeting individuals’ dietary requirements and maintaining normal swallow function for as long as possible. If nutritional requirements could not be met by regular food and fluids, energy rich oral nutritional supplements were prescribed. At Site A, patients attended a multidisciplinary allied health education talk in week one of treatment, followed by weekly dietetic and speech pathology reviews during treatment, and were followed up for a minimum of six weeks post treatment as clinically indicated. At Site B, patients attended a dietitian and speech pathology group education session in week one of treatment. They were then seen on a weekly to fortnightly basis, depending upon nutrition impact symptoms, for up to 6 weeks post treatment. Reactive tubes were utilised at both sites, with Site A using reactive tubes in cases where a prophylactic tube was not placed. Site A used the clinical criteria of oral intake being < 60% of energy requirements, with inadequate intake and no expected improvement for a total of 10 days or more, for the insertion of the reactive feeding tube. In contrast, the decision making regarding reactive feeding tube placement at Site B was made by the radiation oncologist following discussion with the multidisciplinary team members at a case conference or multidisciplinary meeting. At both sites, reactive nasogastric tubes were placed if it was anticipated that a feeding tube would be required at <4 weeks; if the anticipated tube feeding time was >4 weeks, then a reactive gastrostomy would be inserted. Both sites used team consensus and evaluated clinical indicators in collaboration with the patient and family to decide when feeding tubes would be removed.

All data on unplanned admissions and nutrition outcomes were collected prospectively at each site. Nutritional status was assessed at baseline using the validated nutrition assessment tool—the Patient Generated Subjective Global Assessment tool (PG-SGA) [11]. Nutritional status was recorded with a global rating of well-nourished (SGA A), moderate malnutrition (SGA B) or severe malnutrition (SGA C), and the numerical score indicated an increasing risk of malnutrition with a higher score. Body weight (kg) was measured using standard weighing scales at the start of radiotherapy, at the end of the treatment, and again at 4–6 weeks post treatment. Percentage weight loss was calculated from the start to the end of treatment and from the start to 4–6 weeks post treatment, and weight loss outcomes were also classified as ≥5% or ≥10% for each timepoint. An electronic admissions database was used to capture the number of public hospitalisations and the reason(s) for admission as documented by the admitting medical officer. The admissions were classified into planned (elective) and unplanned. An unplanned admission was defined as an unexpected admission or any unplanned prolongation of stay of an elective admission. The reasons for unplanned admissions were then categorised into nutrition-related or other medical reasons (i.e., non-nutrition-related). The classification of these admissions were performed by two dietitians independently reviewing reasons for the admission. If there was no consensus on classification, then the reason for admission was reviewed independently by a third dietitian, with the majority’s decision defining the classification. Nutrition-related admissions were further categorised according to the nutrition impact symptoms as described in the PG–SGA tool. The process of sub-classification followed the same consensus process as described above. The length of stay (days) was also collected for each unplanned admission episode.

Continuous variables and descriptive statistics were presented as mean  ±  standard deviation and median (range). Categorical variables were presented as percentages. The Wilcoxon nonparametric test was used to compare continuous variables between sites whereas the chi-squared test was used to compare differences in categorical variables between the two sites. We fit the binary logistic regression models to identify risk factors for the binary outcomes of nutrition-related admissions (yes/no). All reasonable predictors for nutrition-related unplanned admissions were incorporated in the multivariate logistic model. These explanatory factors included site, age, sex, tumour site, T stage, N stage, radiation dose (Gy), CRT treatment, PG-SGA score, and weight at baseline. To build multivariate logistics models, we used a stepwise procedure with the Akaike Information Criterion (AIC) for model selection. In our descriptive dotplots, we applied LOESS smoothing with a smoothing span of 1.2 for PG-SGA score and 2.0 for weight (kg). All statistical analyses were performed with the statistical package R, version 4.5.0 [12].

## 3. Results

**Figure 2:**
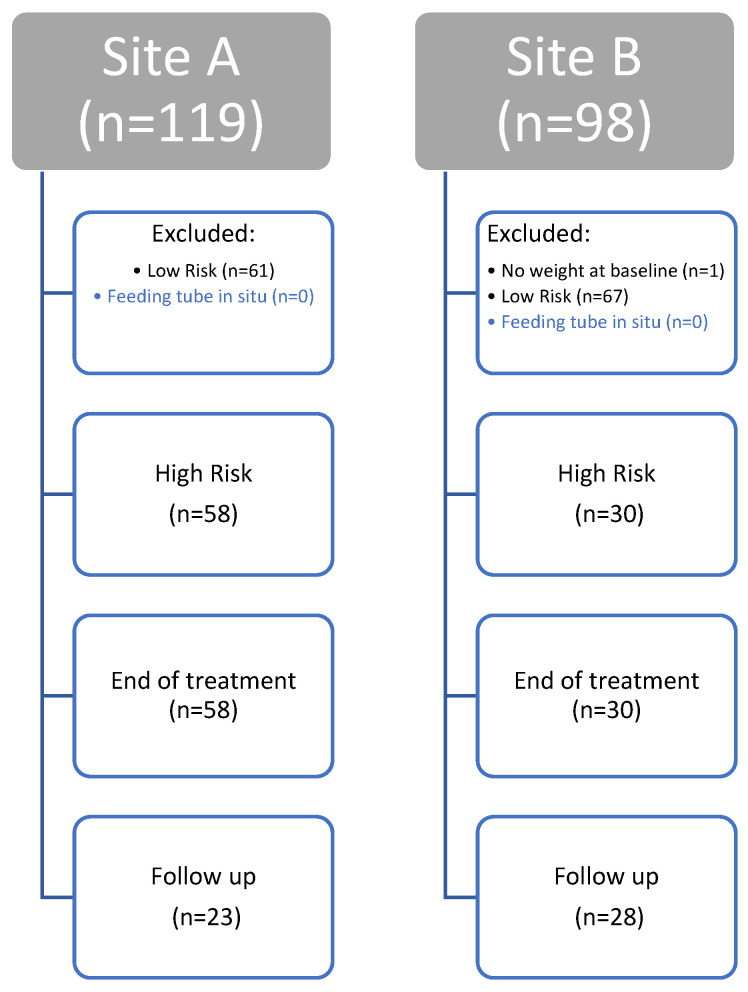
CONSORT diagram to show identification and follow up of eligible patients undergoing curative intent radiotherapy for head and neck cancer.

A total of 88 patients with HNSCC were identified as being eligible for the study (see Figure 2). There were 58 patients from Site A and 30 patients from Site B.

### 3.1. Patient Clinical and Disease Characteristics

The patients’ characteristics are shown in Table 2. The median age at start of radiotherapy was 60 years at both sites (*p* = 0.92). Males accounted for 86% of patients at Site A and 90% of patients at Site B (*p* = 0.74). There were no significant differences between sites with regard to tumour site, type of treatment, radiotherapy fractions or dose, systemic therapy or p16 status. Whilst not statistically significant, there were some clinically important differences in the type of treatments. Site A treated more patients with adjuvant post-operative CRT (*n* = 8, 13.8% vs. *n* = 2, 6.7%, *p* = 0.48), and had more patients treated with weekly cisplatin (*n* = 20, 38% vs. *n* = 6, 20.6%, *p* = 0.38). In contrast, Site B had more patients treated with high dose (HD) cisplatin (*n* = 14, 46.7% vs. *n* = 18, 31%, *p* = 0.38).

### 3.2. Patients’ Nutrition Characteristics

Patients had significantly different mean body weights at baseline (Site A 82 kg vs. Site B 93 kg *p* = 0.004). However, there were no differences in malnutrition risk as measured by PG-SGA (*p* = 0.99). As expected, there were significantly fewer prophylactic tubes placed at Site B compared to Site A (13.3% compared to 62.1% *p* <0.001) (Table 2). This meant that 38% of patients (*n* = 22) at Site A did not have a prophylactic tube as per the guideline recommendation, and of these five went on to have a reactive feeding tube placed. At Site B, eight patients (27%) required a reactive feeding tube.

### 3.3. Weight Loss Outcomes

**Figure 3:**
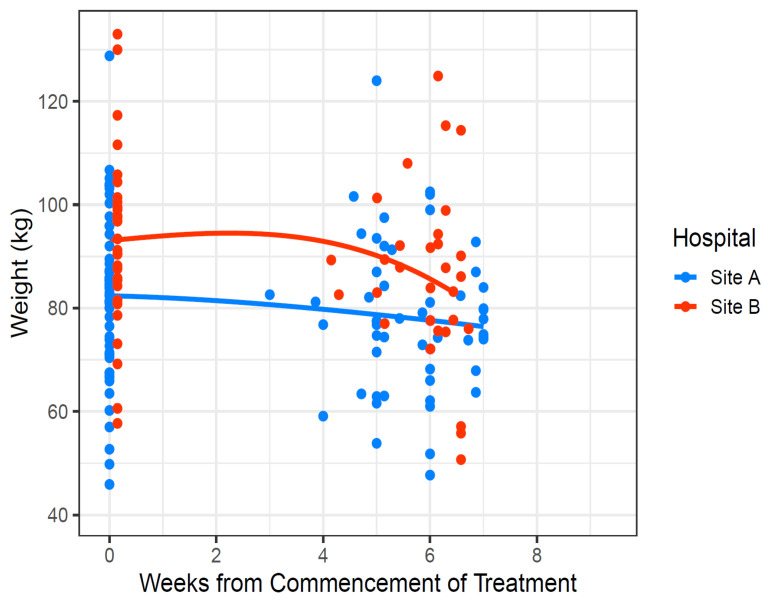
Smoothed scatterplots of weight over the study period by site.

**Figure 4:**
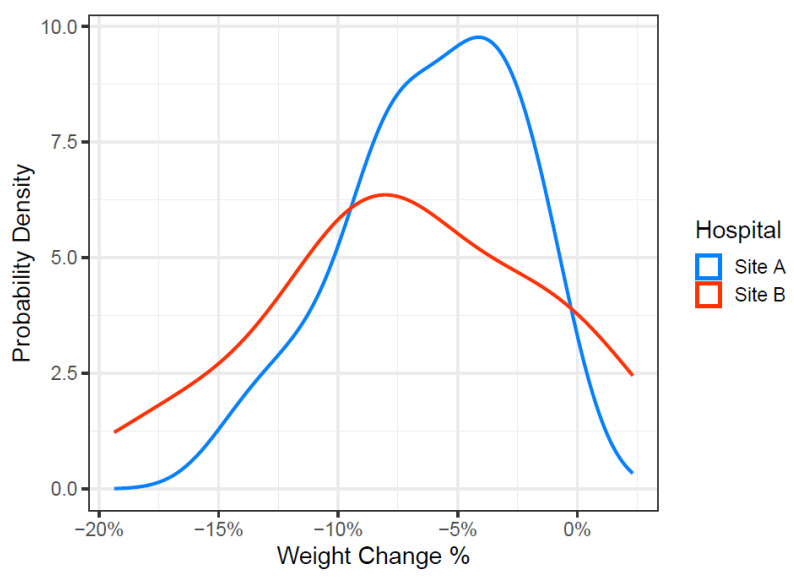
Density plots (smoothed barplots) of percentage weight change during radiotherapy treatment at Site A (blue) and Site B (red).

There was no statistical difference in percentage weight loss across sites at any timepoint (Table 3), although there was a trend for patients at Site B to lose more weight 4–6 weeks post treatment (−9.6% (+/− 5.6%) vs. −11.7% (+/− 6.4%), *p* = 0.150) (Table 4). Site B had slightly heavier subjects at baseline, although the trajectories of weight change were similar across both sites, with a sharper decline from week 4 onwards at Site B (Figure 3). There was also more variability in the weight change percentage among the subjects at Site B (Figure 4).

### 3.4. Unplanned Nutrition-Related Admissions

**Figure 5:**
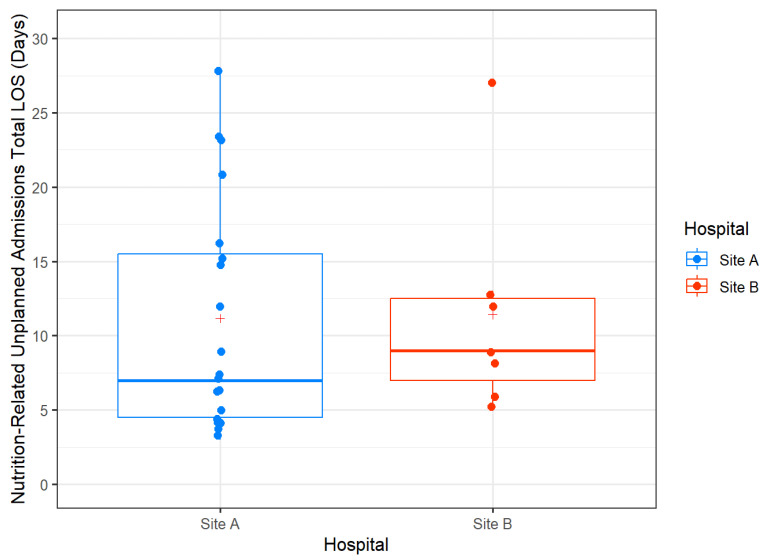
Boxplots of total length of stay (days) by hospital for unplanned nutrition-related admissions for high-risk head and neck cancer patients. Median values are represented by the heavy horizontal line; means are represented by the red +.

Nutrition-related hospital admissions were defined as admissions for toxicities that impacted upon the nutritional status of the patient, with a total of 26 unique patient events. There was no statistical difference across the two sites (Site A, *n* = 19, 32.8%, vs. Site B, *n* = 7, 23.3%; *p* = 0.46). Similarly, the average length of stay for a nutrition-related admission was comparable across the two sites (11.4 (+/− 7.5) days vs. 11.2 (+/−7.9) days; *p* = 0.66) (Figure 5). The most common reasons for nutrition-related admissions were similar across both sites and included nausea, odynophagia, inadequate energy intake, dehydration, and problems with tube feeding (Table 5). Stepwise logistic analysis found that increasing age was a predictor for the risk of a nutrition-related unplanned admission, while conversely a lower dose of radiotherapy reduced the odds of having an unplanned nutrition-related admission (Table 6). The univariate association between unplanned nutrition-related admissions and the variable of definitive radiotherapy versus adjuvant radiotherapy was *p* = 0.48, which indicated that it was not a strong predictor for unplanned nutrition-related admissions and was so this was not included in the final model. Likewise, the univariate associations with unplanned nutrition-related admissions on logistic regression for hospital site was *p* = 0.53 (one degree of freedom), and for tumour site was *p* = 0.49 (three degrees of freedom), and therefore these were not included in the final multivariable model.

### 3.5. Unplanned Medical Admissions

The total number of unplanned admissions for other medical (non-nutrition-related) reasons was six, with a statistically significant difference between sites (Site A, *n* = 1, 1.7%, vs. Site B, *n* = 5, 16.7%; *p* = 0.016). The reasons for unplanned medical admissions included a metabolic disorder, eye infection, allergic reaction, urinary tract infection, otitis media, and non-neutropenic fever.

## 4. Discussion

This prospective multicentre cohort study, in patients with HNSCC deemed at high nutrition risk, compared the clinical and nutrition outcomes obtained with prophylactic versus reactive tube feeding approaches. The study did not find any statistical differences in the primary outcomes of unplanned hospital admissions or the percentage weight loss during and post treatment; however, some clinically important differences were observed.

An international consensus statement of the Academy of Nutrition and Dietetics and the American Society for Parenteral and Enteral Nutrition has defined critical weight loss as involuntary weight loss of ≥5% in 1 month or ≥10% in 6 months [13], and thus the criteria of ≥5% weight loss during chemoradiotherapy treatment has frequently been applied in studies in patients with head and neck cancer [14,15]. Minimising weight loss has been shown to improve treatment tolerance, treatment completion, the rate of emergent care required, and patients’ quality of life [15], and importantly has been shown to improve survival outcomes [16]. Critical weight loss ≥ 5% during treatment has also been associated with poorer survival outcome [17].

Our study found high rates of critical weight loss in both groups, although there were no significant statistical differences in the proportion of high-risk HNSCC patients who had ≥5% weight loss during treatment (*p =* 0.417) or ≥10% weight loss during treatment (*p =* 0.155). The incidence of critical weight loss then continued to rise in the post-treatment period at both sites; again, there were no statistically significant differences between those who had ≥5% weight loss (*p* = 0.182) or ≥10% weight loss (*p =* 0.456). A similar study comparing prophylactic and reactive approaches across two centres in Canada also found no difference in longer-term critical weight loss outcomes (≥10% weight loss) at 1-year post treatment [18]. However, the current study data suggests that the prophylactic feeding approach, compared to the reactive tube feeding approach, did reduce the prevalence of ≥5% critical weight loss both during (from 67% to 55%) and post treatment (from 86% to 70%), and also had similar results for reducing prevalence of ≥10% critical weight loss both during (from 27% to 14%) and post treatment (from 62% to 48%). These findings may have reached statistical significance with a larger sample size and adequate power. This observation has been supported in a number of recent systematic reviews that concluded that prophylactic feeding tubes have result in better nutrition outcomes/less weight loss compared to reactive tube feeding approaches [19,20,21], although one systematic review reported similar nutrition outcomes between the two approaches [22]. McClelland et al. reported the proportion of patients without a prophylactic tube who ultimately receive a reactive tube is highly variable, ranging anywhere from 12% to 72% across the eight studies in their systematic review [19], which is comparable to the 27% we found at Site B in this study.

It is unclear why differences in baseline weights were found between hospital sites in this study. Ethnicity was not collected in this study, which may explain the differences in weight [23]. Site B did have a higher incidence of p16 positive disease (92% vs. 80%), and patients with human papilloma virus (HPV) positive oropharyngeal cancer do tend to be more overweight [24]. Furthermore, patients with HPV-positive cancers are more likely to lose weight [25], and patients with a higher baseline BMI are also at higher risk of losing weight during treatment [26]. Therefore, these factors may have contributed to the higher rates of weight loss seen at Site B.

The prevalence of critical weight loss reported in our study is similar to, if not higher than, other published studies that have reported rates of critical weight loss during treatment in the general HNSCC population to be between 30 and 50% [14,15]. Given that our current study was focused on a subset of HNSCC deemed at high nutritional risk [10], it was not unexpected that our rates of critical weight loss were higher than these mixed HNSCC populations, despite intensive nutrition management strategies through dietary counselling, oral nutrition supplements, and tube feeding. It has been hypothesised that even though patients have prophylactic feeding tubes in situ, the commencement of nutrition support via the tube is often delayed until oral intake declines, or weight loss occurs, thus mirroring a reactive approach. Further prophylactic nutrition support interventions in a randomised controlled trial (RCT) have been studied to address this, with the aim of reducing critical weight loss during treatment, but this approach also did not show any benefits [27]. Patient-related factors were found to be a contributing factor to this negative finding [28], but it has also been suggested that common toxicity criteria and adverse event (CTCAE) scores could be useful to facilitate and guide earlier interventions [29].

Regarding unplanned hospital admission for nutrition-related reasons, our study reported no statistical differences across the two sites (*p* = 0.46), with a similar average length of stay (11.4 days vs. 11.2 days; *p* = 0.66). Although not statistically significant, it was surprising to see the higher number of admissions in the prophylactic group as this was contrary to the results we had seen previously from a similar single centre study at Site A [30] which compared outcomes of proactive versus reactive approaches in the same defined high-risk group. In that study, there was statistically significantly less weight loss (7.0% vs. 9.0%; *p* = 0.048) and fewer unplanned admissions (75% vs. 82%; *p* = 0.029) in the proactive group. This was similar to the trend reported by Olson et al., where they also observed higher admission rates in the reactive group (27% vs. 13%, *p*  =  0.001) [18].

The overall incidence of unplanned admissions in our current study (23–33%) is similar to the literature, with rates reported to be between 22 and 35% [31,32]. Gastrointestinal symptoms (nausea, vomiting, dysphagia, and odynophagia) were identified as the primary reasons for 58–62% of unplanned admissions in the current study. This may highlight the importance of monitoring for these nutrition impact symptoms, as they may contribute to nutrition support targets not being met. Waddle et al. also found that these accounted for 52% of their unplanned admissions [31]. Fahy et al. reported the predominant reasons for unplanned admission being nausea and vomiting (26%), and a general decrease in oral intake/dehydration (30%), with chemoradiotherapy being the only predictor for unplanned admissions [33]. Our multivariate model identified older age as being a factor that increases the risk of an unplanned nutrition-related admission, and thus close monitoring of these higher-risk patient groups is also warranted to implement earlier symptom control and pain management.

The results from this study continue to add to the body of literature in the field. The choice of feeding tube and the optimal timing of its placement has been widely debated in the literature over the last two decades [34,35]. Most of the research which compares prophylactic versus reactive tube feeding has been in retrospective single-centre studies that have significant limitations including sources of selection bias, inconsistency with intervention and outcome definitions, and variations in follow up timeframes [18,36,37,38,39,40,41,42,43,44,45]. Several systematic reviews have failed to make firm conclusions or recommendations due to these limitations of the evidence base [19,20,21,22,46,47,48,49], with all stating a need for more prospective RCT’s to inform definitive conclusions for practice. The systematic review by Mellors et al. specified RCT study design as their inclusion criteria, of which only three studies were identified, reported in five papers [27,50,51,52,53]. In the studies by Brown [27] and Salas [50], all patients had a prophylactic tube placed but the intervention arms used the gastrostomy proactively versus as required. In the RCT by Silander [51,52,53], a prophylactic approach was compared to a reactive approach. The authors of this systematic review concluded with a moderate certainty of evidence, according to the Grading of Recommendations, Assessment, Development, and Evaluation (GRADE), that posited that the prophylactic approach resulted in a clinically important reduction in short-term critical weight loss (>10% weight loss), and significantly improved short-term QoL, but had no other impact on other clinical outcomes [20].

A pilot RCT was also completed in the UK [54] to compare a prophylactic approach with a reactive approach, but the trial did not meet one of the three criteria for progression, as the recruitment rate was lower than hypothesised. A more recent RCT was attempted in India [55], which aimed to compared prophylactic nasogastric tubes with reactive nasogastric tubes. The proportion of patients with significant weight loss at 6 months in the prophylactic versus reactive arms was 33% (*n* = 13) versus 16% (*n* = 10), *p* = 0.44, which suggested some meaningful clinical differences, but the trial was also closed early due to recruitment challenges, limiting interpretation of findings. These recruitment challenges were also identified in an earlier RCT in Australia [56], where patients were randomised to a gastrostomy or nasogastric tube. However, in this trial, both methods had a reactive approach to placement and the initiation of nutrition support, making it difficult to compare to other studies investigating true prophylactic approaches.

The challenges that these RCT’s have all experienced with implementation and recruitment have clearly identified that patient choice and patient preference for the method of tube feeding is critical to their engagement in their healthcare and thus to positive outcomes. A recent prospective study in Thailand [57] aimed to compare a prophylactic gastrostomy approach with oral nutrition support and reactive enteral feeding tube as required. All patients were counselled prior to treatment on the risks and benefits of a prophylactic tube, and the placement of a tube was at the discretion of the patient. The study found better nutrition outcomes in the prophylactic group, but there were no differences in quality of life. Patient counselling plays an important role in helping patients understand why and when a gastrostomy tube may be beneficial or necessary during treatment. To empower patients with their healthcare management it is recommended that clinicians and patients reach a mutual decision on the need and type of tube placement by incorporating clinical factors into decision making while being mindful of patient values [58]. The role of nutrition counselling in a recent systematic review has demonstrated that when delivered as part of multimodal prehabilitation interventions, including in education, oral nutritional supplements, and swallowing exercises, it provides benefits through the maintenance of nutrition intake, body weight, and swallowing function and thus improves quality of life [59]. Therefore, the focus may shift towards enhancing the pre-treatment intervention and education phase for patients and carers to allow informed decision making and consent for their nutrition management [60].

The other important consideration regarding feeding tube placement is related to ensuring that patients maintain oral intake alongside enteral nutrition support, where safe to do so, to minimise swallow muscle atrophy and long-term tube dependence [9]. There are some studies that report that prophylactic tube placement contributes to long-term tube dependency and late dysphagia [61,62]. However, there are some suggestions that maintaining oral intake with an NGT in situ can be more problematic as it can directly interfere with or cause pain and discomfort while eating. Therefore, other studies have reported a nil impact on long-term swallowing problems using the prophylactic tube feeding approach whilst encouraging oral intake [63,64].

A strength of this study was that all eligible high-risk patients were appropriately referred to the dietitian at the commencement of treatment, and thus received access to evidence-based nutritional care, with nil attrition during the treatment phase. However, the patients retained in the follow up were likely the group of patients most at risk, who required the most support, and thus this introduced some potential sample bias in this phase of the study. Some of the loss of follow up post treatment would be due to patients returning to rural/regional/remote areas and accessing local services, and thus is reflective of real-world practice. One of the study’s limitations was that this was a non-randomised study. In addition, as a pragmatic study comparing the overarching models of care in prophylactic versus reactive management, the protocols were not completely standardised across sites for the commencement of reactive tube placement, but reflective of usual care approaches, thus potentially creating some indication and selection bias. However, the key advantage of this multicentre prospective study is the standardised inclusion criteria of high nutrition risk patients only, excluding other non-mucosal head and neck cancers and lymphoma diagnoses, which creates a well-defined homogenous cohort of patients providing a valuable contribution to the literature. While there may have been some inter-site variations in practice, such as the primary method of gastrostomy placement (radiological vs. endoscopic), delivery of chemotherapy regimens (high dose vs. weekly), radiotherapy dose (definitive vs. adjuvant), and hospital-driven criteria or procedures for admissions, these factors (including the hospital site itself) were accounted for in the multivariate model to reduce any confounding effects. A limitation of the analysis was that p16 status could not be adjusted for in the statistical model for unplanned admissions due to the small numbers of the outcome of interest, as the estimates became unstable, and the models did not converge. Finally, it was a relatively small study sample size overall, which lacked the statistical power to adequately compare outcomes between groups, including between those that received surgery and adjuvant radiotherapy, and those that had definitive chemoradiotherapy.

## 5. Conclusions

There were no statistical differences in the rate of unplanned admissions or percentage weight loss outcomes for patients with HNSCC managed under prophylactic versus reactive tube feeding approach. However, the prophylactic approach demonstrated important clinical differences, with less critical weight loss during and post treatment, which may translate into improved clinical and survival outcomes. Decision making regarding the choice of feeding tube should be made in consultation with the patient and the multidisciplinary team, following discussion and education of the risks and known benefits of both approaches.

## Figures and Tables

**Table 1:** Summary of nutrition management strategies and models of care at each Site.

	Site A—Prophylactic Model of Care	Site B—Reactive Model of Care
Criteria for proactive G tube insertion	Oral cavity or oropharyngeal cancer plus bilateral CRT or nasopharyngeal/hypopharyngeal/unknown primary plus CRT or those who were severely malnourished at presentation.	Individual cases for prophylactic tube placement were discussed at the multidisciplinary team (MDT) meeting as per treating team discretion.
Criteria for reactive G tube insertion	Clinical decision making by the multidisciplinary team if oral intake was <60% of energy requirements, with inadequate intake and no expected improvement for a total of 10 days or more and duration of tube feeding anticipated to be >4 weeks.	Clinical decision making by the radiation oncologist following discussion with the multidisciplinary team at a case conference or multidisciplinary meeting and duration of tube feeding anticipated to be >4 weeks.
Criteria for reactive NGT insertion	Clinical decision making by the multidisciplinary team if oral intake was <60% of energy requirements, with inadequate intake and no expected improvement for a total of 10 days or more and duration of tube feeding anticipated to be <4 weeks.	Clinical decision making by the radiation oncologist following discussion with the multidisciplinary team at a case conference or multidisciplinary meeting and duration of tube feeding anticipated to be <4 weeks.
Primary method of G tube placement	Endoscopic placement	Radiological placement
Allied health management during treatment	Multidisciplinary allied health group education talk in week one of treatment, followed by weekly dietetic and speech pathology reviews during treatment.	Dietitian and speech pathology group education talk in week one of treatment, followed by weekly to fortnightly dietetic and speech pathology reviews during treatment, with frequency dependent upon nutrition impact symptoms.
Allied health management post treatment	Dietetic and speech pathology reviews for a minimum of six weeks post treatment, as clinically indicated.	Dietetic and speech pathology reviews for up to 6 weeks post treatment.

**Table 2:** Baseline characteristics of all HNSCC at high risk of requiring at tube at two sites. For categorial data, the data are reported as N (% per site).

Characteristics		Site A *n* = 58	Site B *n*= 30	*p*-Value for Differences
Age (years)	Mean (+/− SD)	60 (+/− 9.6)	59.6 (+/− 8.5)	0.92
Sex	Male %	50 (86.2)	27 (90)	0.74
Tumour Site (%)				0.34
	Oral cavity	8 (13.8)	1 (3.3)	
	Oropharynx	45 (77.6)	24 (80)	
	Nasopharynx	2 (3.4)	2 (6.7)	
	Hypopharynx	3 (5.2)	3 (10)	
Treatment (%)				0.48
	CRT	50 (86.2)	28 (93.3)	
	Post op CRT	8 (13.8)	2 (6.7)	
Radiotherapy Total Fraction	Mean (+/− SD)	34.3 (+/− 1.7)	34.6 (+/− 1.3)	0.46
Dose (Gy)	Mean (+/− SD)	68.6 (+/− 3.4)	69.3 (+/− 2.5)	0.46
Systemic Therapy (%)				0.38
	HD cisplatin	18 (31)	14 (46.7)	
	Weekly cisplatin	20 (38)	6 (20.6)	
	Cetuximab	18 (31)	9 (30)	
	Other	2 (3.4)	1 (3.3)	
T stage (%)				0.82
	T0	2 (3.4)	1 (3.3)	
	T1	10 (17.2)	6 (20)	
	T2	20 (34.5)	10 (33.3)	
	T3	10 (17.2)	6 (20)	
	T4	16 (27.6)	6 (20)	
	Recurrent	0 (0)	1 (3.3)	
N Stage (%)				0.85
	N0	3 (5.2)	1 (3.3)	
	N1	6 (10.3)	5 (16.7)	
	N2	47 (81)	23 (76.7)	
	N3	2 (3.4)	1 (3.3)	
p16 Status				0.56
	Positive	36 (80)	22 (91.7)	
	Negative	6 (13.3)	1 (4.2)	
	Unknown	3 (6.7)	1 (4.2)	
Weight at start of CRT (kg)				0.004
	Mean (+/− SD)	82.4 (+/− 15.5)	93.1 (+/− 17.4)	
PG-SGA Category (%)				1.00
	SGA A	50 (86.2)	24 (85.7)	
	SGA B	7 (12.1)	4 (14.3)	
	SGA C	1 (1.7)	0 (--)	
PG-SGA Score at start CRT				
	Mean (+/− SD)	5 (+/− 3.9)	6.5 (+/− 5.5)	0.99
Type of Tube (%)				<0.001
	Nil	17 (29.3)	18 (60)	
	Proactive G tube	36 (62.1)	4 (13.3)	
	Reactive G tube	1 (1.7)	1 (3.3)	
	Reactive NGT	4 (6.9)	7 (23.3)	

Abbreviation: HNSCC, head and neck squamous cell carcinoma; SD, standard deviation; CRT, chemoradiotherapy, HD, high dose; PG-SGA, patient-generated subjective global assessment; SGA, subjective global assessment; G tube, gastrostomy tube; and NGT, nasogastric tube.

**Table 3:** Number and percentage of high-risk HNSCC patients who had lost ≥ 5% and ≥ 10% of their body weight at the end of radiotherapy and 4–6 weeks post radiotherapy by site.

Weight Loss Outcome	Site A *n* (%)	Site B *n* (%)	*p*-Value for Difference
Weight loss ≥ 5% at the end of treatment	32 (55)	20 (67)	0.417
Weight loss ≥ 10% at the end of treatment	8 (14)	8 (27)	0.155
Weight loss ≥ 5% at 4–6 weeks post treatment	16 (70)	25 (86)	0.182
Weight loss ≥ 10% at 4–6 weeks post treatment	11 (48)	18 (62)	0.456

Abbreviations: HNSCC, head and neck squamous cell cancer.

**Table 4:** Mean percentage weight change in high-risk HNSCC patients from the start until the end of treatment and up to 4–6 weeks post treatment by site.

Weight Change	Site A Mean (+/− SD) Median (Range)	Site B Mean (+/− SD) Median (Range)	*p*-Value for Difference
% weight change at the end of treatment	−6.1 (3.5) −5.6 (−14.2 to −0.8)	−7.2 (5.6) −7.7 (−19.4 to 2.3)	0.350
% weight change at 4–6 weeks post treatment	−9.6 (5.6) −7.9 (−21.8 to −2.5)	−11.7 (6.4) −11.9 (−23.3 to 1)	0.150

Abbreviations: HNSCC, head and neck squamous cell cancer; SD, standard deviation.

**Table 5:** Comparison of causes of nutrition-related unplanned hospital admissions in high-risk HNSCC patients across the two sites.

	Unplanned Admissions in Each Cohort *	
Reason for Admission	Site A (*n* = 19/58)	Site B (*n* = 7/30)
Nausea and vomiting	36% (*n* = 7/19)	29% (*n* = 2/7)
Odynophagia	26% (*n* = 5/19)	29% (*n* = 2/7)
Dehydration	16% (*n* = 3/19)	29% (*n* = 2/7)
Poor oral intake	16% (*n* = 3/19)	14% (*n* = 1/7)
Problems with tube feeding	5% (*n* = 1/19)	0% (*n* = 0/7)

Abbreviations: HNSCC, head and neck squamous cell cancer. * Admission data represents unique patient events rather than unique events.

**Table 6:** Stepwise logistic analysis (AIC) to find multivariate predictors for nutrition-related unplanned admissions in high-risk HNSCC patients.

Term	Estimate	SE	t	P	OR	OR 95% CI Lower	OR 95%CI Upper
(Intercept)	8.908	5.255	1.695	0.090	-	-	-
Age	0.060	0.030	2.041	0.041	1.062	1.002	1.125
Radiation dose	−0.200	0.081	2.465	0.014	0.819	0.698	0.960

Abbreviations: AIC, Akaike Information Criterion; HNSCC, head and neck squamous cell cancer; SE, standard error; OR, odds ratio; and CI, confidence interval.

## Data Availability

Data supporting reported results can be requested via the corresponding author.

## Abbreviations

The following abbreviations are used in this manuscript:

HNSCC	Head and neck squamous cell cancer
LOS	Length of stay
MDT	Multidisciplinary team
CRT	Chemoradiotherapy
H-IMRT	Helical intensity-modulated radiotherapy
PGSGA	Patient Generated Subjective Global Assessment
SGA	Subjective Global Assessment
HD	High dose
SD	Standard deviation
NGT	Nasogastric tube
HPV	Human papilloma virus
BMI	Body mass index
RCT	Randomised controlled trial
CTCAE	Common toxicity criteria for adverse events
QOL	Quality of life
GRADE	Grading of recommendations, assessment, development and evaluation
